# Antiphospholipid antibodies in pulmonary embolism treated with direct oral anticoagulants: Prevalence data from unselected consecutive patients

**DOI:** 10.1016/j.rpth.2023.100050

**Published:** 2023-01-18

**Authors:** Tummas Justinussen, Jorgen Brodersen Gram, Mustafa Vakur Bor

**Affiliations:** 1 Department of Clinical Biochemistry, Thrombosis and Anticoagulation Clinic, University Hospital of Southern Denmark, Esbjerg, Denmark; 2 Department of Regional Health Research, Unit for Thrombosis Research, University of Southern Denmark, Esbjerg, Denmark

**Keywords:** anti-β2 glycoprotein I autoantibody, anticardiolipin antibodies, anticoagulants, antiphospholipid syndrome, pulmonary embolism, thrombosis

## Abstract

**Background:**

Direct oral anticoagulants (DOACs) are the first-choice treatment option for the prevention of the recurrence of venous thrombosis in patients with pulmonary embolism (PE); however, their effect in patients with antiphospholipid syndrome (APS) is challenged. Therefore, the prevalence of antiphospholipid autoantibodies in patients with PE is noteworthy.

**Objectives:**

To determine the prevalence of unselected patients presenting with PE who meet the criteria for APS based on elevated levels of anticardiolipin (aCL) and anti-β2-glycoprotein I (aβ2GPI) antibodies.

**Methods:**

Consecutive patients with PE, in whom DOACs were primarily initiated, were tested for aCL and aβ2GPI. If the levels were elevated, the tests were repeated after 12 weeks for APS diagnosis. Laboratory results and patient characteristics were retrospectively collected from a laboratory information system and electronic patient journal entries over a 2-year period.

**Results:**

The prevalence of APS based on consistently elevated levels of aCL or aβ2GPI was 3.7% (10 of 267 patients). Three patients were double positive. In 11 out of 21 patients (52%) with initially elevated values, the levels of the antibodies normalized after 12 weeks. The patient characteristics were largely similar in those with APS and those without APS; however, patients with APS tended to be older and more likely to receive antithrombotic treatment at the time of PE.

**Conclusion:**

We found a relatively low prevalence of APS based on aCL or aβ2GPI. The high rate of normalized levels of the antibodies after 12 weeks reaffirms the need for repeated tests for APS diagnosis. Older patients more frequently met the criteria for APS. Determining the effectiveness of DOACs in non–triple-positive patients with APS following venous thromboembolism is important to further determine the feasibility of unselected tests in patients with PE.

## Essentials

• The rate of antiphospholipid syndrome in patients with pulmonary embolism (PE) is ill defined.
• This was a retrospective report of consecutive patients with PE from a thrombosis treatment clinic.
• The elevated levels of anticardiolipin or anti-β2-glycoprotein I antibodies normalized in 11 of 21 patients.
• A relatively low prevalence of antiphospholipid syndrome (3.7%) was detected in unselected patients with PE.

## 1 Introduction

Pulmonary embolism (PE) ranks high among cardiovascular causes of mortality, and treatment with anticoagulants after the event is a cornerstone in the prevention of long-term recurrence and morbidity [1]. Current guidelines recommend direct oral anticoagulants (DOACs) as first-choice anticoagulation therapy after PE in most patients [1,2].

Antiphospholipid syndrome (APS) is characterized by an increased risk of venous or arterial thrombosis and/or obstetric complications in patients who have persistently elevated levels of autoantibodies against phospholipid (aPL)-binding proteins. According to the Sydney classification criteria, for definitive diagnosis of APS, elevated levels of anti-β2-glycoprotein I (aβ2GPI), anticardiolipin (aCL), and/or lupus anticoagulants (LAs) should be documented after a clinical event in 2 tests separated by at least 12 weeks [3]. In thrombotic APS, vitamin K antagonists (VKAs) are generally indicated in place of DOACs as secondary prophylaxis [4,5]. Unselected testing for aPL antibodies after PE is not the usual practice. However, the potential treatment implications of APS make attention to the frequency of elevated levels of aPL antibodies in patients presenting with PE relevant.

This retrospective report examined the prevalence of APS based on consistently elevated levels of aβ2GPI and aCL antibodies in unselected patients with PE in whom DOACs were primarily initiated.

## 2 Methods

### 2.1 Patient population

Patients diagnosed with PE at the University Hospital of Southwestern Denmark are referred to a thrombosis and anticoagulation clinic for follow-up within a month after their acute hospital course. As part of the diagnostic evaluation and to support decision making regarding thrombosis prophylaxis, all patients are tested for aβ2GPI and aCL antibodies at the first visit to the clinic as a routine protocol. If the levels are elevated, they are retested after a minimum period of 12 weeks to diagnose APS. LAs are not tested because of the risk of confounded results in these patients, in whom treatment has begun, primarily with DOACs, during their hospital stay. While evaluating whether patients should undergo further thrombophilia tests, in addition to those for aβ2GPI and aCL, the team reviews the presentation of the patient with adherence to Danish recommendations for thrombophilia workup.

### 2.2 Blood sampling and analysis

The methods for blood sampling, sample prehandling, and biochemical analyses are described in detail elsewhere [6]. aCL and aβ2GPI analyses were performed using EliA (Thermo Fisher Scientific), fully automated random access fluorescence enzyme immunoassays on the Phadia 250 instrument (Phadia GmbH), with a coefficient of variation of <10% for both the antibodies. A 99th percentile cutoff was used, as is the recommended practice [3,7], determined by in-house validation in keeping with the approach suggested by the International Society on Thrombosis and Haemostasis (ISTH) [6].

### 2.3 Data collection and presentation

Patient records were determined eligible for review if they were referred to the clinic between December 1, 2019, and November 8, 2022, with registration of PE diagnosis codes (DI26, DI260, DI260A, DI269, and DI269) in the International Statistical Classification of Diseases and Related Health Problems, 10th Revision, classification system. To minimize the potential for missing patients with PE who were inadvertently not registered with electronic diagnostic codes, the list produced by the registered codes was cross-referenced with a physical booking list. The booking list was kept by the clinic nurses, who cataloged patient diagnosis and the status of blood sampling for aPL antibodies.

The results of aβ2GPI and aCL tests were obtained from the laboratory information system. Relevant patient characteristics and risk factors, as presented in Table 1, were obtained via manual review by the main author of electronic patient journal entries on admission to the emergency department, based on description of PE diagnosis by a radiologist, and at the first visit to the thrombosis and anticoagulation clinic. Finally, conclusions from team conferences were obtained.

**Table 1:** Characteristics of patients with and without antiphospholipid syndrome.^a^^,^^b^

Characteristic	Patients with antiphospholipid syndrome (*n* = 10)	Patients without antiphospholipid syndrome (*n* = 257)
General characteristics		
Age (y)	81.5 (16.5)	69.0 (20.0)
BMI (kg/m^2^)	26.2 (23.9-28.7)	28.1 (27.4-28.9)
Women	4 (40)	125 (49)
Double-positive APS	3 (30)	NA
Time from PE to aPL test (d)	21 (5)	16 (15)
Time from PE to start of data collection (d)	406 ± 176	400 ± 214
Acute diagnosis and treatment		
Diagnostic modality – CT^c^	7 (70)	175 (68)
Central embolus	3 (30)	81 (32)
Thrombolysis	0 (0)	8 (3)
Treatment with DOAC	8 (80)	219 (85)
VTE risk profile		
Autoimmune disease	2 (20)	43 (17)
Active cancer	3 (30)	40 (16)
Previous cancer	2 (20)	30 (12)
Receiving chemotherapy	1 (10)	14 (6)
Familial disposition (venous or arterial)	0 (0)	59 (23)
History of previous VTE	1 (10)	47 (18)
Estrogen therapy at diagnosis	0 (0)	6 (2)
Antithrombotic therapy at diagnosis^d^	6 (60)	69 (27)
COVID-19 illness as a possible risk factor	0 (0)	10 (4)
COVID-19 vaccine as a possible risk factor	0 (0)	9 (4)
PE interpreted as unprovoked	7 (70)	133 (52)
Additional cardiovascular risk profile		
Smoking^e^	4 (40)	114 (44)
Antihypertensive therapy at diagnosis	8 (80)	125 (49)
Antilipid therapy at diagnosis	3 (30)	90 (35)
Antidiabetic therapy at diagnosis	0 (0)	28 (11)
History of previous arterial thrombosis	3 (30)	54 (21)
Pulmonary hypertension on the first echocardiography^f^	6 (60)	101 (39)
Atrial fibrillation	1 (10)	26 (10)
Additional workup and treatment		
Patients selected for full thrombophilia panel testing^g^	1 (10)	48 (19)
VKA chosen as a substitute for DOAC at confirmed APS	10 (100)	NA
Indefinite prophylactic treatment planned	10 (100)	115 (45)

aPL, autoantibodies against phospholipid; APS, antiphospholipid syndrome; BMI, body mass index; CT, computed tomography; DOAC, direct oral anticoagulant; PE, pulmonary embolism; VKA, vitamin K antagonist; VTE, venous thromboembolism.

a Data presented as mean ± SD, geometric mean (95% CI), median (interquartile range), or number (percentage).

b Statistical comparisons were not performed because of the risk of type I error.

c CT included PE found using CT pulmonary angiography and incidentally using abdominal CT (*n* = 2) or positron emissions tomography CT (*n* = 5). All others were detected using scintigraphy.

d Five patients with APS on antiplatelet therapy and 1 on VKAs. Fifty-one patients without APS on antiplatelet therapy alone, 3 on double antiplatelet therapy, 1 on antiplatelet plus low-molecular–weight heparin therapy, 1 on low-molecular–weight heparin alone, and 1 on VKA.

e Currently smoking or previously cumulated 10 years with 20 cigarettes/d.

f Tricuspid regurgitation gradient of >30 mm Hg or, when not specified, a conclusion of “increased pulmonary pressure” in the operator’s notes.

g The patient with APS was heterozygous for factor V Leiden mutation. Four patients without APS had high-risk congenital thrombophilia (1 had compound heterozygous for factor V Leiden and prothrombin mutations, 1 had antithrombin deficiency, and 2 had protein S deficiency), and 6 were heterozygous for factor V Leiden mutation.

The regional council approved the study for access to data records (Journal Number 21/49334). According to Danish guidelines, this registry-based study was not subject to review by an ethics board.

Continuous variables are presented as mean ± SD when normally distributed, geometric mean (95% CI) when natural logarithmic transformation was used, and otherwise as median (interquartile range) when skewed. Categorical variables are reported as numbers and proportions (%).

## 3 Results and Discussion

We identified 287 consecutive patients with PE for review, either using the diagnostic codes (248 patients) or via cross-reference to booking lists (39 patients). A total of 18 patients were excluded because of incorrect registering of diagnostic codes (*n* = 4), because of reversal of diagnosis after review of uncertain radiologic findings (*n* = 3), or because they were not tested for the aPL antibodies (*n* = 11).

In sum, 269 patients were tested for the aPL antibodies. Two patients who were positive for the aPL antibodies did not undergo repeat measurement at 12 weeks, both because of comorbidities (severe dementia and disseminated cancer) and a lack of a clinical consequence of repeat measurement. They were excluded from the APS versus non-APS comparisons. Of the resulting patients, a total of 257 did not meet the criteria for APS, ie, they had a negative aPL antibody test result the first time or, when the level was elevated in the first test, had normalized values when retested after a minimum of 12 weeks. Thus, 10 patients met the Sydney criteria for APS based on elevated levels in 2 tests, resulting in a prevalence of APS of 3.7% (Table 1). The detailed characteristics of patients with APS are presented in Table 2. Three were double positive (2 with aβ2GPI and aCL immunoglobulin [Ig]G and 1 with aβ2GPI and aCL IgM), and 7 were single positive (2 with aCL IgG, 2 with aβ2GPI IgG, and 3 with aβ2GPI IgM). The first patient in Table 2 showed a noticeable drop in her values between the baseline and confirmatory measurements. Additional measurement would be of interest to determine whether aPL levels remain elevated; however, this was not performed within the sampling period.

**Table 2:** Detailed characteristics of patients who met the criteria for antiphospholipid syndrome.

Sex	Age (y)	Elevated values at the first test (type, titer)^a^	Elevated values at the second test 12 wk later (type, titer)^a^	Autoimmune disease	PE interpreted as provoked by	Previous thrombosis	Additional thrombophilia tests
Female	63	aCL IgG, 279 aβ2GPI IgG, 470	aCL IgG, 23 aβ2GPI IgG, 48	None	Unprovoked	2 arterial	Not tested
Female	74	aCL IgG, 101 aβ2GPI IgG, 156	aCL IgG, 109 aβ2GPI IgG, 192	None	Unprovoked	None	Not tested
Female	88	aCL IgM, 37^b^ aβ2GPI IgM, 129	aCL IgM, 78 aβ2GPI IgM, 53	SLE	Unprovoked	1 VTE and 1 arterial	Not tested
Male	47	aCL IgG, 26	aCL IgG, 23	None	Unprovoked	None	Factor V Leiden heterozygous
Male	69	aβ2GPI IgM, 16	aβ2GPI IgM, 18	None	Active cancer and intensive care^c^	None	Not tested
Male	81	aβ2GPI IgG, 59	aB2GPI IgG, 55	None	Active cancer	1 arterial	Not tested
Male	82	aβ2GPI IgG, 11	aβ2GPI IgG, 19	None	Unprovoked	None	Not tested
Male	86	aβ2GPI IgM, 16	aβ2GP IgM, 16	None	Active cancer and pneumonia^c^	None	Not tested
Male	87	aβ2GPI IgM, 22	aβ2GPI IgM, 21	None	Unprovoked	None	Not tested
Female	93	aCL IgG, 29	aCL IgG, 26	PMR	Unprovoked	None	Not tested

aβ2GPI, anti-β2-glycoprotien I; aCL, anticardiolipin; APS, antiphospholipid syndrome; Ig, immunoglobulin; PE, pulmonary embolism; PMR, polymyalgia rheumatic; SLE, systemic lupus erythematosus; VTE, venous thromboembolism.

a The 99th percent cutoff values were 11 AU/mL for aβ2GPI IgG, 12 AU/mL for aβ2GPI IgM, 21 GPL-U/mL for aCL IgG, and 41 MPL-U/mL for aCL IgM.

b Not elevated.

c Cancer workup was done in parallel with APS testing, and the management of coexistent APS and cancer was performed in a case-by-case approach.

Overall, those who met the criteria for APS and those who did not were similar, apart from patients with APS being older and more frequently on antithrombotic treatment at the time of PE (Table 1). Although the latter finding may be driven by greater thrombotic potential in older patients, previous studies have also indicated a relatively high frequency of recurrence in patients with APS despite antithrombotic treatment [8]. One patient with APS had systemic lupus erythematosus, and 1 had polymyalgia rheumatica. In patients without APS, the relative frequency of autoimmune diseases was similar (20% vs 17%). One had systemic lupus erythematosus, and the other disease groups were autoimmune arthritic diseases (*n* = 13, not arthritis urica), autoimmune pulmonary diseases (*n* = 13, not asthma), autoimmune kidney diseases (*n* = 4), inflammatory bowel diseases (*n* = 3), and others (*n* = 9).

DOACs were initiated in most patients (34% on apixaban, 44% on rivaroxaban, and 7% on edoxaban vs 11% on low-molecular–weight heparin and 4% on VKAs). The treatment of thrombotic APS with DOACs remains a debated topic [9]. In a recent meta-analysis by Khairani et al. [10], which included 4 randomized controlled trials, with 472 patients, a 5-fold increased odds ratio for subsequent arterial thrombosis was reported in patients randomized to DOACs versus that in those randomized to treatment with VKAs after thrombotic APS. No difference was reported for subsequent venous thrombosis or for major bleeding. A subgroup analysis according to the type of APS (triple positive vs other combinations) did not show evidence of effect modification in the meta-analysis. Rivaroxaban was the subject of study in 3 randomized controlled trials and apixaban in 1. In view of these results, some guidelines discourage the use of DOACs in all patients with APS [1], whereas others only strongly recommend VKAs in favor of DOACs in those with triple-positive APS or with arterial events and vary in their approach to the treatment of non–triple-positive patients with venous thrombosis [9]. The addendum to the guidelines for the management of APS from the British Society of Hematology suggests that DOACs should be continued over no anticoagulation therapy if patients do not wish to switch to VKAs [5]. The ISTH guidelines on the use of DOACs in patients with thrombotic APS have noted in their guidance statements that discussion and shared decision making could be applied to patients with non–“high-risk” APS, who have already had several months of good adherence to treatment with DOACs after the first episode of venous thromboembolism [4]. Because of these key knowledge gaps, further research has been encouraged in patients with “lower-risk” APS [4]. In our report, all patients with APS switched to long-term treatment with VKAs following a team conference–based consensus and dialogue with the patients.

In a review of the literature on APS in 2013, Andreoli et al. [11] reported the frequency of aPL antibodies in 9.5% of patients with deep venous thrombosis across 21 studies, which resembles our report of 8.6% of patients with initial positivity for aPL antibodies. In 3 more recent retrospective studies, definitive APS was reported in ∼4% to 10% of patients with venous thromboembolism selected for thrombophilia workup [[12], [13], [14]]. With the exclusion of patients who were single positive for LAs in these studies, the results adjust down to ∼3% to 8% of patients with APS based on elevated levels of aCL and aβ2GPI antibodies, more comparable with our findings.

Six out of 10 patients who met the criteria for APS were aged >80 years (Table 2). It could be challenged whether elderly patients without any medical history of autoimmune disease or previous thrombosis should be considered to have newly developed, clinically relevant APS despite meeting the criteria. Studies have reported a high aPL positivity in older patients [[15], [16], [17], [18]]. However, the findings on clinical presentation in older versus younger patients with APS differ because some have found higher rates of primary APS and triple aPL positivity in older patients [16,17], and others have reported higher rates of single-positive APS [18]. Male predominance has also been reported to be more prevalent in older patients [16,18]. Because of the lack of evidence on APS in this subgroup, the optimal treatment option is unknown for older patients with consistently elevated values. Our cutoffs were determined in a local healthy population aged between 18 and 66 years. The possibility that an added positive correlation between age and normal aPL titers could play a role in our findings cannot be ruled out. There are no age-specific cutoffs, and interestingly, those used by our laboratory were more rigorous than those set by the manufacturer (>10 U/mL for positive results in all Phadia/EliA aPL analyses set by the manufacturer vs 99th percentile cutoff values of 11 AU/mL for aβ2GPI IgG, 12 AU/mL for aβ2GPI IgM, 21 GPL-U/mL for aCL IgG, and 41 MPL-U/mL for aCL IgM determined in house [6]. If the cutoffs recommended by the manufacturer are applied, the number of patients with elevated aPL levels during the first test increases from 23 to 41, driven by more cases with aCL IgG (16 vs 8 patients) and aCL IgM (18 vs 1 patient), and 7 patients shift from being single positive to double positive. This further highlights the impact while choosing a cutoff value.

In clinical guidelines, it is suggested to classify elevated titers of aCL and aβ2GPI determined using enzyme-linked immunosorbent assay into “low range” (20-40 “units”) or “moderate-to-high range” (>40 “units” or >99th percentile) [19,20]. Our 99th percentile Phadia or EliA cutoffs, excluding that for aCL IgM, were lower in absolute terms than the 40-unit thresholds; however, as previously reported by Vandevelde et al. [21], the agreement of these thresholds, when applied to automated systems, as is used in this report, can be poor. Furthermore, while taking into consideration the potential variability in titers among platforms, our locally derived 99th percentile, which has also been suggested by the ISTH guidelines, should provide the best estimate for our population.

Although unselected patients with a confirmed diagnosis of PE, repeated aPL tests after 12 weeks, and a locally derived 99th percentile cutoff value determined with adherence to recommended guidelines [3,6,7] are the strengths of this report, the omission of LA testing is an important limitation. The omission of LA tests prevents us from registering patients with single-LA positivity and those with triple-positivity APS, often characterized as “high risk,” presumably causing underestimation of APS. The concurrent use of anticoagulants made reliable LA testing inaccessible in our setting; however, the application of alternative procedures, such as DOAC-stop, could provide a future perspective on LA testing in patients undergoing treatment with active DOACs [22]. Screening based on lupus-sensitive activated partial thromboplastin time when patients are admitted and prior to starting treatment with DOACs may also help identify patients who would benefit from LA testing. The implementation of this would, however, require increased communication and coordination with admitting clinicians at the emergency department. The lack of specific information on the race and ethnicity of the patients is another limitation. Data evaluating whether race and ethnicity affect the prevalence of APS are scarce; however, few studies have suggested that they play a role [23,24]. All our participants were from a single center in Denmark and were expected to be predominately Caucasian. Thus, our findings may not be generalizable across all ethnicities.

In conclusion, we reported a prevalence of 3.7% for APS based on elevated levels of aCL or aβ2GPI antibodies in unselected patients with PE, in whom DOACs were primarily initiated. Older patients more frequently met the criteria for APS. The high rate of normalized antibody levels after 12 weeks (52%) reaffirms the need for repeated tests for APS diagnosis.

## References

[1] Konstantinides S.V., Meyer G., Bueno H., Galié N., Gibbs J.S.R., Ageno W. (2020). 2019 ESC guidelines for the diagnosis and management of acute pulmonary embolism developed in collaboration with the European Respiratory Society (ERS). Euro Heart J.

[2] National Institute for Health and Care Excellence. Venous thromboembolic diseases: diagnosis, management and thrombophilia testing. NG158. March 26, 2020. Available from: https://www.nice.org.uk/guidance/ng158.

[3] Miyakis S., Lockshin M.D., Atsumi T., Branch D.W., Brey R.L., Cervera R. (2006). International consensus statement on an update of the classification criteria for definite antiphospholipid syndrome (APS). J Thromb Haemost.

[4] Zuily S., Cohen H., Isenberg D., Woller S.C., Crowther M., Dufrost V. (2020). Use of direct oral anticoagulants in patients with thrombotic antiphospholipid syndrome: guidance from the Scientific and Standardization Committee of the International Society on Thrombosis and Haemostasis. J Thromb Haemost.

[5] Arachchillage D.R., Gomez K., Alikhan R., Anderson J.A., Lester W., Laffan M. (2020). Addendum to British Society for Haematology Guidelines on Investigation and Management of Antiphospholipid syndrome, 2012 (*Br J Haematol*. 2012;157:47-58): use of direct acting oral anticoagulants. Br J Haematol.

[6] Bor M.V., Jacobsen I.L., Gram J.B., Sidelmann J.J. (2018). Revisiting the Phadia/EliA cut-off values for anticardiolipin and anti-β2-glycoprotein I antibodies: a systematic evaluation according to the guidelines. Lupus.

[7] Devreese K.M., Pierangeli S.S., De Laat B., Tripodi A., Atsumi T., Ortel T.L. (2014). Testing for antiphospholipid antibodies with solid phase assays: guidance from the SSC of the ISTH. J Thromb Haemost.

[8] Cervera R., Serrano R., Pons-Estel G.J., Ceberio-Hualde L., Shoenfeld Y., De Ramón E. (2015). Morbidity and mortality in the antiphospholipid syndrome during a 10-year period: a multicentre prospective study of 1000 patients. Ann Rheum Dis.

[9] Pastori D., Menichelli D., Cammisotto V., Pignatelli P. (2021). Use of direct oral anticoagulants in patients with antiphospholipid syndrome: a systematic review and comparison of the international guidelines. Front Cardiovasc Med.

[10] Khairani C.D., Bejjani A., Piazza G., Jimenez D., Monreal M., Chatterjee S. (2022). Direct oral anticoagulants vs vitamin-K antagonists in thrombotic antiphospholipid syndrome: meta-analysis of randomized controlled trials. J Am Coll Cardiol.

[11] Andreoli L., Chighizola C.B., Banzato A., Pons-Estel G.J., De Jesus G.R., Erkan D. (2013). Estimated frequency of antiphospholipid antibodies in patients with pregnancy morbidity, stroke, myocardial infarction, and deep vein thrombosis: a critical review of the literature. Arthritis Care Res.

[12] Miranda S., Park J., Le Gal G., Piran S., Kherani S., Rodger M.A. (2020). Prevalence of confirmed antiphospholipid syndrome in 18-50 years unselected patients with first unprovoked venous thromboembolism. J Thromb Haemost.

[13] Martinelli I., Abbattista M., Bucciarelli P., Tripodi A., Artoni A., Gianniello F. (2018). Recurrent thrombosis in patients with antiphospholipid antibodies treated with vitamin K antagonists or rivaroxaban. Haematologica.

[14] Ho W.K., Rigano J. (2018). Prevalence of antiphospholipid antibodies in an Australian cohort of patients with unprovoked venous thromboembolism. Pathology.

[15] Goldman-Mazur S., Wypasek E., Karpiński M., Stanisz A., Undas A. (2019). High detection rates of antithrombin deficiency and antiphospholipid syndrome in outpatients aged over 50 years using the standardized protocol for thrombophilia screening. Thromb Res.

[16] Belizna C., Latino O., Stojanovich L., Saulnier P., Devreese K., Udry S. (2020). Should be older patients tested for antiphospholipid antibodies? 695 cases from the retrospective series HIBISCUS [abstract]. Ann Rheum Dis.

[17] Grimaud F., Yelnik C., Pineton De Chambrun M., Amoura Z., Arnaud L., Costedoat Chalumeau N. (2019). Clinical and immunological features of antiphospholipid syndrome in the elderly: a retrospective national multicentre study. Rheumatol.

[18] Crosnier C., Dufrost V., Audrain M., Hemont C., Agard C., Connault J. (2022). Syndrome des antiphospholipides après 65 ans: étude comparative rétrospective des caractéristiques clinicobiologiques et des récidives thrombotiques [abstract]. La Rev Médecine Interne.

[19] Garcia D., Erkan D. (2018). Diagnosis and management of the antiphospholipid syndrome. N Engl J Med.

[20] Sayar Z., Moll R., Isenberg D., Cohen H. (2021). Thrombotic antiphospholipid syndrome: a practical guide to diagnosis and management. Thromb Res.

[21] Vandevelde A., Chayoua W., de Laat B., Gris J.C., Moore G.W., Musiał J. (2022). Semiquantitative interpretation of anticardiolipin and antiβ2glycoprotein I antibodies measured with various analytical platforms: communication from the ISTH SSC Subcommittee on Lupus Anticoagulant/Antiphospholipid Antibodies. J Thromb Haemost.

[22] Platton S., Hunt C. (2019). Influence of DOAC Stop on coagulation assays in samples from patients on rivaroxaban or apixaban. Int J Lab Hematol.

[23] Manzano-Gamero V., Pardo-Cabello A.J., Vargas-Hitos J.A., Zamora-Pasadas M., Navarrete-Navarrete N., Sabio J.M. (2018). Effect of ethnicity on clinical presentation and risk of antiphospholipid syndrome in Roma and Caucasian patients with systemic lupus erythematosus: a multicenter cross-sectional study. Int J Rheum Dis.

[24] Niznik S., Rapoport M.J., Avnery O., Ellis M.H., Hajyahia S., Agmon-Levin N. (2022). Ethnicity and antiphospholipid syndrome in Israel. Arthritis Care Res.

